# Ceftazidime-avibactam as monotherapy or in combination for targeted treatment of KPC-producing *Klebsiella pneumoniae* infections in ICUs: a comparative analysis through counterfactual framework and desirability of outcome ranking

**DOI:** 10.1007/s10096-026-05529-x

**Published:** 2026-05-05

**Authors:** Andrea Marino, Alberto Enrico Maraolo, Maria Mazzitelli, Alessandra Oliva, Nicholas Geremia, Andrea De Vito, Federica Cosentino, Chiara Gullotta, Vincenzo Scaglione, Eleonora Vania, Sara Lo Menzo, Paolo Navalesi, Lorenzo Volpicelli, Andrea Fiori, Pamela Prestifilippo, Annamaria Cattelan, Claudio Maria Mastroianni, Giordano Madeddu, Bruno Cacopardo, Giuseppe Nunnari

**Affiliations:** 1 Department of Clinical and Experimental Medicine, Infectious Diseases Unit, ARNAS Garibaldi Hospital, University of Catania, Catania, Italy; 2 Section of Infectious Diseases, Department of Clinical Medicine and Surgery, University of Naples “Federico II”, Naples, Italy; 3 Dipartimento di Sicurezza e Bioetica, Sezione di Malattie Infettive, Università Cattolica del Sacro Cuore, Rome, Italy; 4 Department of Medical and Surgical Sciences, Fondazione Policlinico Universitario Agostino Gemelli IRCCS, Rome, Italy; 5 Infectious and Tropical Diseases Unit, Padua University Hospital, Padua, Italy; 6 Department of Public Health and Infectious Diseases, Sapienza University of Rome, Rome, Italy; 7 Unit of Infectious Diseases, Department of Clinical Medicine, Ospedale “dell’Angelo”, Venice, Italy; 8 Unit of Infectious Diseases, Department of Medicine, Surgery and Pharmacy, University of Sassari, Sassari, Italy; 9 Department of Medicine (DIMED), Institute of Anesthesia and Intensive Care, University of Padua, Padua University Hospital, Padua, Italy; 10 School of Medicine, University of Sassari, Sassari, Italy; 11 Intensive Care Unit, ARNAS Garibaldi Hospital, Catania, Italy

**Keywords:** Ceftazidime-avibactam, DOOR, Propensity score, Inverse probability of treatment weighting, CRE, Bloodstream infections, Pneumonia

## Abstract

**Purpose:**

To evaluate the causal effect of ceftazidime/avibactam (C/A) combination therapy versus monotherapy on mortality and clinical success in patients with KPC-producing *Klebsiella pneumoniae* (KPC-Kp) infections in intensive care unit.

**Methods:**

This multi-centre, retrospective observational study (2021–2023) included adults with KPC-Kp bloodstream infections or pneumonia treated with C/A-based regimens. We employed a counterfactual framework using inverse probability of treatment weighting (IPTW) to estimate the average treatment effect on 30-day mortality. Clinical success was further assessed using Desirability of Outcome Ranking (DOOR) analysis and partial credit scoring based on patient-perspective scenarios.

**Results:**

Among 123 included patients, 77 (62.6%) received monotherapy and 46 (37.4%) received combination therapy. The combination group presented with significantly higher baseline severity, including higher APACHE II scores and rates of septic shock. In the IPTW-adjusted analysis, 30-day survival was 73.8% (95% CI: 56–92%) with combination therapy compared with 60.8% (95% CI: 46.8–77%) with monotherapy. The survival probability ratio was 1.21 (95% CI: 0.80–1.45), indicating no statistically significant survival benefit. The DOOR analysis showed a 54.7% (95% CI: 48.9%–60.4%) probability of a more favourable outcome with combination therapy, which was not statistically significant. Mean partial credit scores did not differ significantly across scenarios prioritizing survival or adverse event avoidance.

**Conclusions:**

In this cohort, C/A-based combination therapy did not provide a significant survival advantage or an improved clinical desirability ranking compared with monotherapy, after adjusting for confounding factors.

**Supplementary Information:**

The online version contains supplementary material available at 10.1007/s10096-026-05529-x.

## Introduction

The global dissemination of carbapenem-resistant Enterobacterales (CRE), particularly *Klebsiella pneumoniae* carbapenemase (KPC)-producing *K. pneumoniae* (KPC-Kp), represents a critical challenge in intensive care units (ICUs) due to limited therapeutic options and high associated mortality rates [1–4]. For nearly a decade, ceftazidime-avibactam (C/A) has served as a cornerstone of therapy, demonstrating superior clinical efficacy compared to older, more toxic “best available” regimens such as colistin or aminoglycosides [5].

However, as clinical experience with C/A has expanded, significant debate persists regarding the optimal treatment strategy [6]. Specifically, whether C/A should be administered as monotherapy or in combination with other agents—such as carbapenems, tigecycline, or aminoglycosides—remains a point of contention among clinicians [7]. While some early observational data suggested potential synergy and reduced risk of resistance emergence with combination therapy [8], current expert consensus, including the 2024 Infectious Diseases Society of America (IDSA) guidance, generally recommends C/A monotherapy for KPC-producing isolates [9].

Recent literature reflects this ongoing controversy. A multicenter analysis found that C/A combination therapy might offer a short-term (14-day) survival advantage in specific high-risk subgroups, such as those with severe pneumonia [10]. Conversely, several other recent cohorts and a network meta-analysis have reported no significant difference in 30-day mortality between monotherapy and combination regimens [11, 12]. These conflicting findings are often confounded by “confounding by indication,” where more severely ill patients are more likely to receive combination therapy, thereby obscuring the true causal effect of the treatment.

To address these limitations, this study utilizes a counterfactual framework and Inverse Probability of Treatment Weighting (IPTW) to evaluate the causal effect of C/A-based combination therapy versus monotherapy in critically ill patients with KPC-Kp infections. Furthermore, acknowledging that survival alone may not capture the full clinical picture, we employ the Desirability of Outcome Ranking (DOOR) analysis. This innovative approach provides a more comprehensive assessment of clinical success by integrating mortality, clinical relapse, and adverse events into a single, patient-centered metric.

## Methods

### Study design and setting

This was a multicentre, retrospective, observational cohort study involving five ICUs from five cities in Italy (Catania, Sassari, Roma, Padova, and Venezia). The study cohort comprised patients treated during a 36-month period from January 2021 through December 2023.

### Participants

At each participating centre, clinical teams identified potentially eligible patients using institutional databases and medical record reviews. Inclusion criteria comprised consecutive adults (≥ 18 years) diagnosed with bloodstream infection (BSI) or pneumonia caused by *Klebsiella pneumoniae* carbapenemase (KPC)–producing strains (KPC-Kp) who received targeted therapy with C/A for at least 48 h. Dose selection was left to the discretion of the treating physicians. Patients were followed for up to 30 days from the date of the index culture. Exclusion criteria included invasive infections other than BSI or pneumonia, receipt of less than 48 h of study drugs due to death within the first 48 h and isolates resistant to the study drug.

### Clinical variables and definitions

Demographic, clinical, and microbiological data were retrospectively collected from hospital medical records. Collected variables included age, sex, and body mass index (BMI), as well as comorbidities and severity of underlying conditions, assessed using the Charlson Comorbidity Index [13].

Severity of illness at infection onset was evaluated using the Sequential Organ Failure Assessment (SOFA) score [14] and the Acute Physiology and Chronic Health Evaluation II (APACHE II) score [15].

KPC-producing *Klebsiella pneumoniae* (KPC-Kp) bloodstream infection (BSI) was defined by isolation of KPC-Kp from blood cultures (BCs) in the presence of clinical signs of infection; BSI onset corresponded to the date of collection of the index BC.

BSI and pneumonia were defined by the presence of at least one positive set of BCs and radiological findings consistent with pneumonia accompanied by compatible clinical signs, symptoms, and laboratory parameters, respectively [16].

Pneumonia was defined by the presence of a new or progressive radiographic infiltrate on chest X-ray or CT scan, accompanied by at least two clinical criteria (fever > 38 °C or hypothermia < 36 °C, leukocytosis or leukopenia, purulent secretions, or worsening oxygenation), and microbiological confirmation via semi-quantitative cultures of respiratory specimens (endotracheal aspirate ≥ 10⁵ CFU/mL or BAL ≥ 10⁴ CFU/mL) yielding KPC-Kp as the predominant pathogen. All diagnoses underwent attending physician adjudication at each site. VAP was defined according to onset timing relative to mechanical ventilation, consistent with established clinical criteria.

Combination therapy was defined as the administration of two or more antimicrobial agents for a minimum duration of 48 h.

Companion agents were selected by the treating physicians according to local microbiological information and clinical judgement. Because of the retrospective multicentre design, detailed agent-specific susceptibility data for adjunctive drugs and dosing information for all companion agents were not systematically retrievable for every case.

The appropriateness of empirical therapy—defined as antimicrobial treatment initiated before availability of susceptibility results—was assessed when KPC-Kp isolates were susceptible to C/A and when the agent was administered within 48 h from index blood culture collection.

Early (within 48–72 h) clinical improvement was defined as at least one of the following: weaning from vasopressors; fever disappearance > 48 h; procalcitonin reduction by > 80%; and C-reactive protein reduction by > 75% [8].

### Microbiological studies

Bacterial isolates were identified according to standard laboratory procedures. In accordance with the routine protocols of the participating hospitals’ microbiology laboratories aimed at expediting diagnostic workflows, bacterial pellets obtained from positive blood cultures (BCs) were directly used for identification by matrix-assisted laser desorption/ionization time-of-flight mass spectrometry (MALDI-TOF MS; Bruker Daltonics). Detection of the blaKPC gene was subsequently performed using the GeneXpert^®^ system (Cepheid). Antimicrobial susceptibility testing was conducted according to local laboratory workflows using either the VITEK 2 automated system (bioMérieux, Marcy l’Étoile, France) or the Sensititre™ system (Thermo Fisher Scientific). Susceptibility interpretation followed the contemporaneous EUCAST breakpoints in use at the participating Italian centres during the study period. Because of the retrospective multicentre design, the exact method used for individual agents such as colistin and fosfomycin could not be uniformly reconstructed for all cases. The short time to active therapy observed in the cohort likely reflects the routine use of rapid identification from positive blood cultures by MALDI-TOF MS together with rapid blaKPC detection by GeneXpert at participating centres.

### Outcomes

The primary outcome was all-cause mortality, assessed at 30-day from treatment beginning, in the framework of a time-to-event analysis. Relapses, defined as new onset of signs and symptoms of infection occurring after initial clinical improvement and microbiological cure, necessitating a new course of active antibiotic therapy, were assessed as well.

### Statistical analysis

Descriptive statistics were calculated, with continuous variables summarized as means with standard deviations or medians with interquartile ranges, according to their distribution. Normality was assessed with Shapiro-Wilk test. Continuous variables were compared using the Student’s t-test or Wilcoxon rank-sum test, as appropriate. Categorical variables were expressed as frequencies and percentages and compared using the chi-squared test or Fisher’s exact test, as appropriate. Statistical significance was defined as a two-sided p-value < 0.05.

We employed a counterfactual framework to estimate the causal effect of combination antibiotic therapy (ceftazidime-avibactam [C/A] plus additional agent) compared to monotherapy (C/A alone) on 30-day mortality. The analysis assumed positivity, counterfactual consistency, no-interference (stable unit treatment value assumption - SUTVA) and conditional exchangeability [17]. Average treatment effects (ATEs) were estimated to provide population-level contrasts, which are more generalizable than conditional effects and less sensitive to model misspecification [18].

To address confounding, we applied inverse probability of treatment weighting (IPTW) to create a balanced pseudo-population [19]. Propensity scores were estimated using logistic regression including baseline covariates: age, sex, body mass index ≥ 30 kg/m², comorbidities (chronic obstructive pulmonary disease, chronic kidney disease, diabetes mellitus, cardiovascular disease, onco-hematologic disease, cirrhosis), APACHE II score at infection onset, infection acquisition setting, mechanical ventilation, extracorporeal membrane oxygenation, renal replacement therapy, infection type, polymicrobial nature, and rectal colonization status. Although APACHE II conceptually incorporates age and chronic health components, individual comorbidities were retained in the propensity score model because they capture treatment-allocation-relevant pathophysiology distinct from the composite score. Covariate balance was assessed using standardized mean differences (SMDs), with values > 0.10 indicating substantial imbalance. Propensity scores were trimmed at the 1st and 99th percentiles to ensure overlap, and weights were stabilized for efficiency [19].

A Cox proportional hazards model was fitted to the weighted pseudo-population with time to death or censoring as the outcome. The model included stabilized IPTW weights and was adjusted for all baseline covariates to improve precision. Continuous covariates were transformed using natural splines with 2 degrees of freedom, with internal knots at quartiles, to account for non-linearity [20]. Partial effects plots visualized non-linear relationships for age and APACHE II score. Collinearity was assessed using adjusted Generalized Variance Inflation Factor (aGVIF), with values > 2 indicating concern.

Since Cox models yield conditional hazard ratios rather than marginal causal estimands, we computed marginal survival curves via g-computation [21]. Model-based predictions were averaged across the weighted population to obtain marginal survival probabilities with 95% confidence intervals estimated via bootstrapping. Survival probability ratios (SPRs) were calculated at 7, 14, and 30 days [22]. Stratified sensitivity analyses by infection type assessed robustness. An additional pre-specified sensitivity analysis restricted the combination arm to patients receiving C/A plus fosfomycin (*n* = 29) versus monotherapy (*n* = 77), given the predominance of this companion agent. A further sensitivity analysis included time to active treatment as an additional covariate in the model.

A Desirability of Outcome Ranking (DOOR) analysis provided comprehensive assessment beyond mortality [23]. The three-level ordinal scale ranked outcomes as: (1) alive at 30 days without relapse; (2) alive with relapse at 30 days; (3) dead at 30 days. Within the IPTW-weighted pseudo-population, we fitted weighted proportional odds logistic regression to estimate the odds of achieving better DOOR rankings with combination therapy. Sequential dichotomization analyzed each threshold separately. Partial credit analysis evaluated three hypothetical patient-perspective scenarios, as previously described [24]: Scenario A (survival-centric), Scenario B (event-free prioritization), and Scenario C (balanced clinical utility). Mean partial credit scores were compared between groups, with 95% CIs crossing zero indicating no significant difference.

Additional methodological details, including assumptions, propensity score specification, weight diagnostics, and DOOR implementation, are provided in the Supplementary Appendix (Supplementary Material).

#### Software

All analyses were performed using R version 4.4.3 (R Foundation for Statistical Computing). Propensity score estimation and IPTW calculation were conducted using the *Weightit* package; survival and causal inference analysis employed the *survival and* the *marginaleffects* package, respectively. Utilizing the free online application at https://methods.bsc.gwu.edu/ [23], we executed the DOOR analysis. Statistical significance was considered in case of *p* ≤ 0.05.

### Ethics and approval

This study was conducted in accordance with the Declaration of Helsinki and received approval from the local Ethics Committees (Protocols: 101/CECT2, 0069/2022, and 0341/2023). Given the retrospective study design, the requirement for informed consent was waived.

## Results

### Study population and baseline characteristics

A total of 123 patients were included in the study, with 77 (62.6%) receiving C/A monotherapy and 46 (37.4%) receiving C/A combination therapy. The median age was comparable between groups (64.0 vs. 66.0 years, *p* = 0.863); however, the combination group had a significantly higher proportion of male patients (65.2% vs. 37.7%, *p* = 0.006).

Patients in the combination therapy group presented with a significantly more acute clinical profile. These patients had higher median APACHE II scores (18.5 vs. 18.0, *p* = 0.005) and required higher rates of organ support, including mechanical ventilation (65.2% vs. 32.5%, *p* = 0.001) and dialysis (32.6% vs. 9.1%, *p* = 0.002). Septic shock was also more prevalent in the combination group (52.2% vs. 28.6%, *p* = 0.015).

Baseline comorbidities also varied significantly; while chronic kidney disease was more prevalent in the monotherapy group (31.2% vs. 13.0%, *p* = 0.041), the combination group had higher rates of onco-hematologic malignancies (23.9% vs. 5.2%, *p* = 0.005) and cardiovascular disease (47.8% vs. 24.7%, *p* = 0.015).

Regarding infection characteristics, the monotherapy group consisted entirely of BSI (100%), whereas the combination group included cases of pneumonia alone (19.6%) and polymicrobial infections (34.8% vs. 16.9%, *p* = 0.041).

Notably, the monotherapy group was significantly more likely to have received active empirical treatment (80.5% vs. 54.3%, *p* = 0.004). Total treatment duration was longer in the combination group (median 12.0 days [IQR 8.0–17.8]) compared with monotherapy (median 10.0 days [IQR 7.0–12.0]; *p* = 0.015), and patients in combo group also experienced a significantly higher rate of clinical relapse (17.4% vs. 3.9%, *p* = 0.027).

Among patients receiving combination therapy, the most frequently administered companion regimen was fosfomycin (29/46, 63.0%). Other companion agents included aminoglycosides (6/46, 13.0%), tigecycline (5/46, 10.9%), and colistin (2/46, 4.3%). Because of the retrospective multicentre design, detailed adjunctive-agent susceptibility data, exact tigecycline dosing, and species-level characterization of co-pathogens in polymicrobial infections were not uniformly available across centres and were therefore not analyzed separately. Table 1 shows the baseline characteristics.

In terms of C/A infusion modality, administration differed significantly between groups (*p* < 0.001). Continuous infusion was more frequently used in the monotherapy group (47/77, 61.0%) than in the combination group (11/46, 23.9%). Conversely, intermittent infusion (17/46, 37.0% vs. 9/77, 11.7%) and extended infusion (18/46, 39.1% vs. 21/77, 27.3%) were more commonly adopted among patients receiving combination therapy.

In terms of secondary clinical/microbiological outcomes, early improvement was comparable between groups (46/77, 59.7% in monotherapy vs. 28/46, 60.9% in combination therapy; *p* = 1.000). Candidemia occurred more frequently in the combination-therapy group (7/46, 15.2%) than in the monotherapy group (3/77, 3.9%), although this difference did not reach statistical significance (*p* = 0.060). Conversely, relapse was significantly higher among patients receiving combination therapy (8/46, 17.4%) compared with monotherapy (3/77, 3.9%; *p* = 0.027). Clostridioides difficile infection was uncommon, with no events in the monotherapy group and one case in the combination-therapy group (0/77, 0.0% vs. 1/46, 2.2%; *p* = 0.794).

**Table 1:** Baseline characteristics between treatment groups. BMI: body mass index; COPD: chronic obstructive pulmonary disease; CKD: chronic kidney disease; DM: diabetes mellitus; MV: mechanical ventilation; TPN: total parenteral nutrition; CVC: central venous catheter; PICC: peripherally inserted central catheter; BSI: bloodstream infection; *KPC rectal colonization at the moment of infection. Deep vascular access was defined as the presence of any central vascular device (including CVC, PICC, or PORT-A-CATH). “Midline” indicates a peripherally inserted catheter with tip terminating in a proximal upper-extremity vein and not in the central venous system. Intermittent infusion: each dose administered over 2 h; extended infusion: each dose administered over 3–4 h; continuous infusion: the total daily dose administered as a 24-hour infusion after a loading dose

Variables			
	Monotherapy	Combination therapy	*p*
n	77	46	
Demographic information			
Age (median [IQR])	64.00 [55.00, 75.00]	66.00 [55.50, 74.75]	0.863
Gender male, n(%)	29 (37.7)	30 (65.2)	0.006
BMI ≥ 30, n(%)	21 (27.3)	14 (30.4)	0.865
COPD, n(%)	12 (15.6)	7 (15.2)	1.000
CKD, n(%)	24 (31.2)	6 (13.0)	0.041
Oncohematology diseases, n(%)	4 (5.2)	11 (23.9)	0.005
DM, n (%)	25 (32.5)	10 (21.7)	0.285
Heart disease, n (%)	19 (24.7)	22 (47.8)	0.015
Cirrhosis, n (%)	5 (6.5)	1 (2.2)	0.520
Conditions at baseline			
APACHE II (median [IQR])	18.00 [18.00, 18.00]	18.50 [16.00, 26.00]	0.005
Death at 30-day, n (%)	15 (19.5)	15 (32.6)	0.155
Time to active treatment (median [IQR]), days	1.00 [0.00, 1.00]	1.00 [0.00, 2.00]	0.989
Treatment duration (median [IQR]), days	10.00 [7.00, 12.00]	12.00 [8.00, 17.75]	0.015
Community-acquired, n (%)	2 (2.6)	1 (2.2)	1.000
Hospital acquired, n (%)	72 (93.5)	40 (87.0)	0.365
MV, n (%)	25 (32.5)	30 (65.2)	0.001
ECMO, n (%)	2 (2.6)	2 (4.3)	0.997
TPN, n (%)	37 (48.1)	30 (65.2)	0.096
Dialysis, n (%)	7 (9.1)	15 (32.6)	0.002
Deep vascular access, n (%)	77 (100.0)	46 (100.0)	NA
Arterial line, n (%)	24 (100.0)	35 (83.3)	0.089
CVC, n (%)	50 (64.9)	38 (82.6)	0.058
PICC or midline, n (%)	20 (26.0)	13 (28.3)	0.947
PORT-A-CATH, n (%)	3 (3.9)	1 (2.2)	1.000
KPC-rectal-colonization, n (%)*	74 (96.1)	38 (82.6)	0.027
Sepsis, n (%)	74 (96.1)	40 (87.0)	0.127
Septic Shock, n (%)	22 (28.6)	24 (52.2)	0.015
Infection type (%)			< 0.001
BSI, n(%)	77 (100.0)	37 (80.4)	
Pneumonia alone n(%)	0 (0.0)	9 (19.6)	
BSI plus pneumonia, n(%)	63 (81.8)	16 (34.8)	
BSI alone, n(%)	14 (18.2)	21 (45.7)	
Polymicrobial infection, n(%)	13 (16.9)	16 (34.8)	0.041
Infusion modality (%)			< 0.001
Intermittent, n(%)	9 (11.7)	17 (37.0)	
Extended, n(%)	21 (27.3)	18 (39.1)	
Continuous, n(%)	47 (61.0)	11 (23.9)	
Empirical treatment (of any type), n (%)	62 (80.5)	32 (69.6)	0.244
Empirical active treatment, n (%)	62 (80.5)	25 (54.3)	0.004
Combo fosfomycin, n (%)	0 (0.0)	29 (63)	< 0.001
Combo aminoglycoside, n (%)	0 (0.0)	6 (13.0)	0.005
Combo colistin, n (%)	0 (0.0)	2 (4.3)	0.268
Combo tigecycline, n (%)	0 (0.0)	5 (10.9)	0.013

### Counterfactual framework analysis

Covariate balance after IPTW is shown in Supplementary Fig. 1, demonstrating how the procedure minimized imbalances among groups. In Supplementary Fig. 2 partial effect plots regarding age and APACHE II score, modelled taking into account potential non-linearity, are depicted. Formal multicollinearity assessment using aGVIF confirmed no covariate exceeded the threshold of 2 (Supplementary Table 1), supporting model specification.

**Fig. 1:**
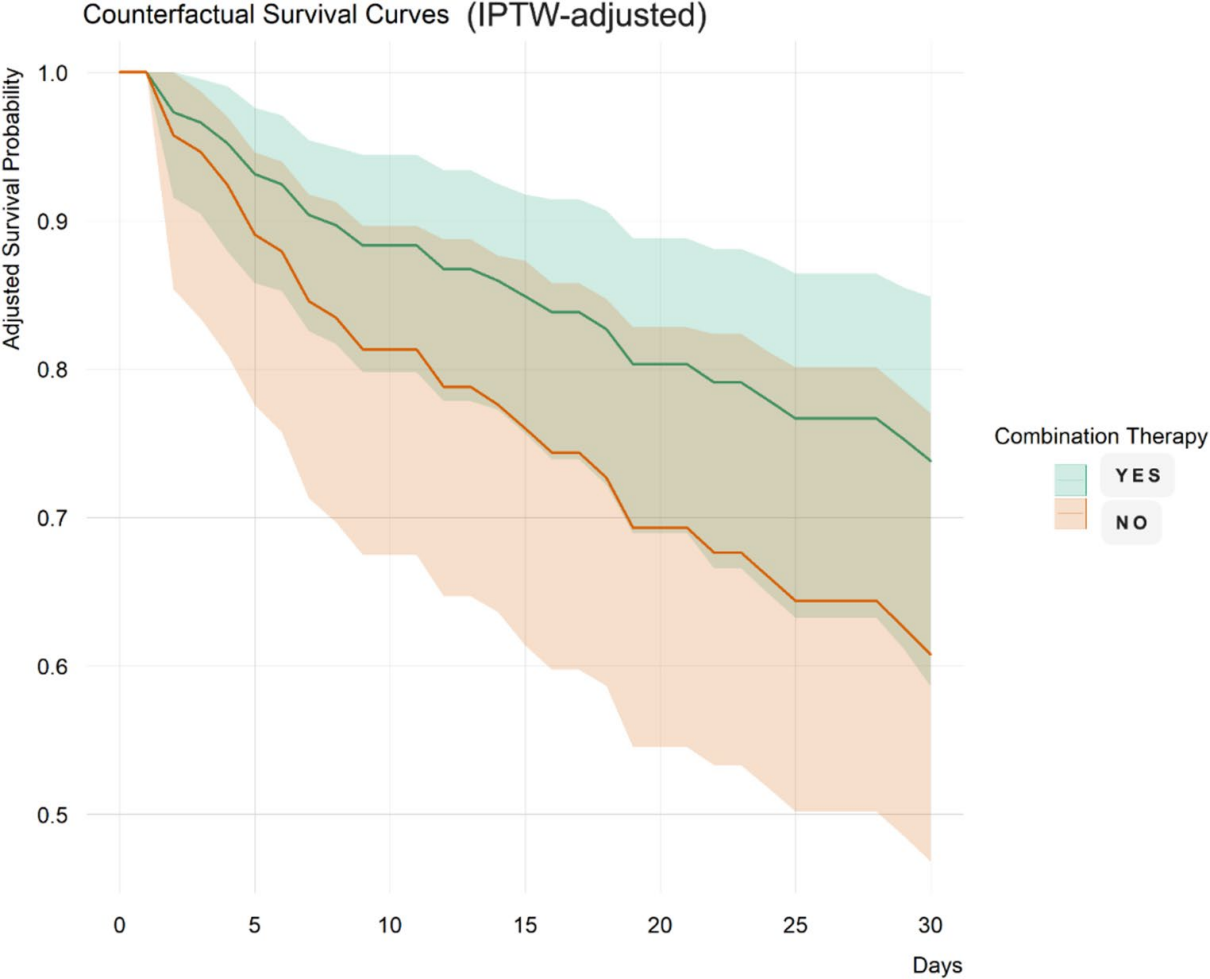
Counterfactual adjusted survival curves after inverse probability of treatment weighting (IPTW) in the overall population

Marginal counterfactual survival probabilities were estimated via g-computation and plotted as adjusted curves (Fig. 1). Absolute differences in survival probability between combination therapy and monotherapy were small at all time points and associated with substantial uncertainty, with confidence intervals spanning the null value. No clinically meaningful survival benefit was observed with combination therapy over monotherapy (Table 2). At the primary endpoint of 30 days, adjusted survival was 73.8% (95% CI: 56–92%) for combination therapy compared to 60.8 (95% CI: 46.8–77%) for monotherapy (SPR = 1.21; 95% CI: 0.80–1.45). Data at previous timepoints are reported in the Table 2.

**Fig. 2:**
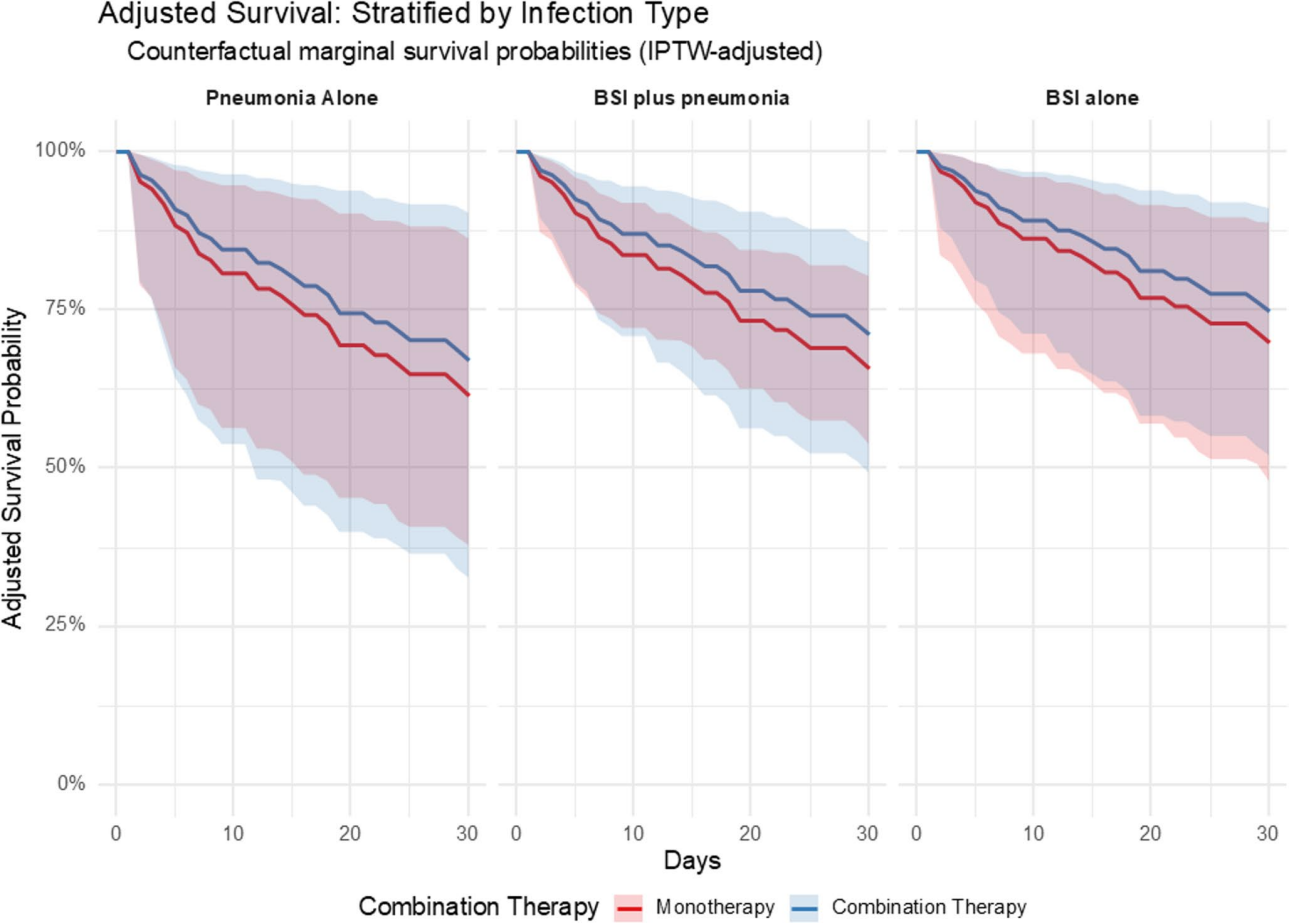
Counterfactual adjusted survival curves after inverse probability of treatment weighting (IPTW) in the overall population and stratification according to infection type the pneumonia-only stratum contains no patients in the monotherapy arm. Survival estimates for this subgroup are model-derived and should be interpreted as exploratory only; the positivity assumption cannot be considered satisfied for this stratum

To assess treatment effect heterogeneity, we stratified the analysis by infection type. The overlapping confidence intervals across strata suggest no clear evidence of treatment effect modification (Fig. 2). Specifically, SPRs between combination and monotherapy were 1.03 (95% CI: 0.57–1.45) in the subgroup of pneumonia cases, 1.02 (95% CI: 0.81–1.22) in the subgroup of BSI plus pneumonia and 1.02 (95% CI: 0.83–1.20) also in the one of BSI alone. Especially in the pneumonia sub-cohort stratified estimates were imprecise due to smaller sample size and event counts. The survival estimates for this stratum are derived entirely from model-based extrapolation within the weighted framework, rather than from directly observed comparisons and should be interpreted as exploratory only.

**Table 2:** Survival probability ratios (SPR)

Time (Days)	Survival Probability (%) Combination therapy	Survival Probability (%) Monotherapy	Survival probability ratio (SPR)Estimate	2.5% (Lower Bound)	97.5% (Upper Bound)
**7**	90.4	84.6	1.07	0.78	1.21
**14**	86	77.6	1.11	0.70	1.25
**30**	73.8	60.8	1.21	0.80	1.45

In a sensitivity analysis restricted to the C/A + fosfomycin subgroup versus monotherapy, the IPTW-adjusted 30-day survival probability was 70.8% (fosfomycin) versus 61.4% (monotherapy), corresponding to a SPR of 1.15 (95% CI: 0.74–1.42), which did not reach statistical significance (Supplementary Table 2). When time to active treatment was added to the model, the magnitude and direction of the results did not materially change, confirming the absence of a statistically significant effect, although 30-day survival estimates were lower in both groups (Supplementary Table 3).

### Desirability of outcome ranking analysis

**Fig. 3:**
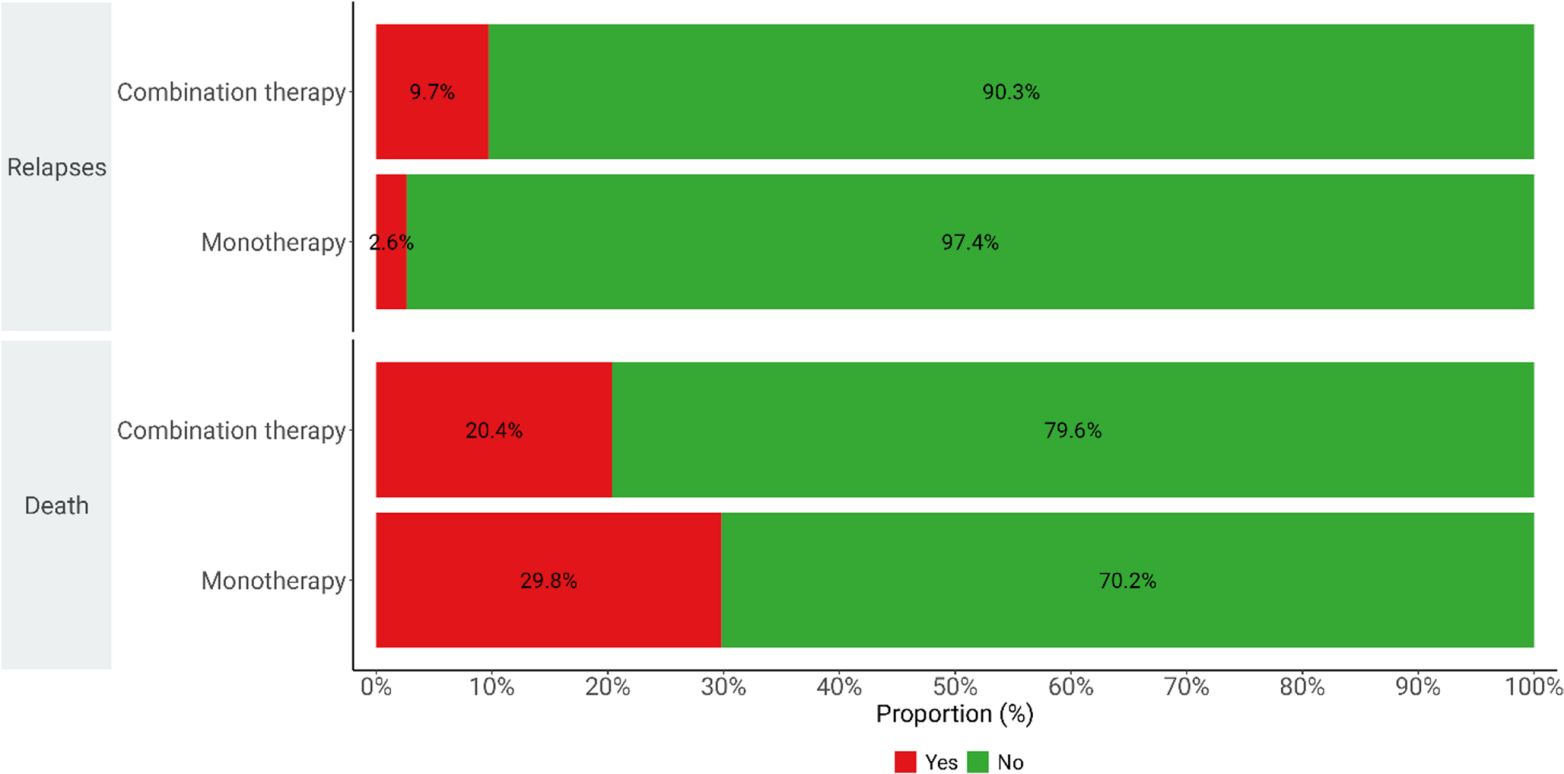
Comparison of desirability of outcome ranking (DOOR) distribution between combination therapy and monotherapy with ceftazidime-avibactam in the overall study population after inverse probability of treatment weighting (IPTW)

Figure 3 displays the distribution of DOOR rankings for the weighted pseudo-population (217 “weighted” observations based on 123 patients). Specifically, the number of deaths was lower in the combination arm (20.4%) compared to the monotherapy arm (29.8%). On the contrary, the weighted frequency of alive patients with relapse was higher in the combination group (9.7% vs. 2.6%). In Supplementary Fig. 3 DOOR probabilities according to each outcome are presented.

**Fig. 4:**
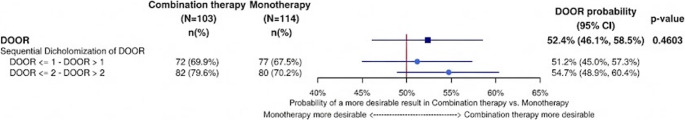
Forest plot of the confidence interval estimates for the cumulative desirability of outcome ranking (DOOR) probability based on sequential dichotomization of the DOOR outcome. Forest plot displaying the probability that a randomly selected patient receiving combination therapy achieves a more favourable DOOR outcome than a randomly selected patient receiving monotherapy, estimated through sequential dichotomization of the three-level DOOR scale. Each row represents a threshold: ‘> dead’ (i.e., probability of being alive) and ‘> alive with relapse’ (i.e., probability of being alive without relapse). Estimates were derived from weighted proportional odds logistic regression in the IPTW pseudo-population. Values > 50% favour combination therapy; 95% confidence intervals crossing 50% indicate no statistically significant difference

The adjusted analysis indicated that the benefit linked with combination therapy was not statistically significant: when sequentially dichotomizing outcomes (Fig. 4), the probability of being alive without relapse was higher in the combination group (51.2%) but with CI crossing the line of null effect (95% CI: 45%-57.3%), and the same applied to the probability of being alive with or without relapse, resulting the combination therapy more desirable but not in statistically significant fashion (54.7%, 95% CI: 48.9%–60.4%). 

Table 3 details the comparison of DOOR partial credit scores, based on three different scoring frameworks assigning full, no, or partial credit to each DOOR category (Supplementary Table 4). In all scenarios no significant difference in mean partial credit scores was found. The highest difference was detected in scenario A, the one placing more value on hospital survival): 9.4, but with p value equal to 0.109 (95% CI: -2.1–21.0)

**Table 3:** Desirability of outcome ranking (DOOR) partial credit analysis in overall study population. ^a^ To evaluate outcomes based on varying patient priorities, three distinct frameworks were applied: Scenario **A**: Focuses exclusively on hospital survival (binary mortality). Scenario **B**: Prioritizes successful discharge to home without any adverse outcomes. Scenario **C**: Balances the importance of survival against the desire to avoid specific complications. For each scenario, we calculated the mean partial credit scores for both treatment groups and determined the between-group difference. Results were considered statistically non-significant if the 95% CI for the difference included zero

Scenarios^a^	Mean combination score	Mean monotherapy score	Differences in DOOR score	95% CI	*P*-value
Scenario A	79.6	70.2	9.4	(-2.1, 21.0)	0.109
Scenario B	69.9	67.5	2.4	(-10.1, 14.8)	0.710
Scenario C	76.7	69.4	7.3	(-4.2, 18.8)	0.210

## Discussion

In this multicentre cohort of critically ill ICU patients with KPC-Kp bloodstream infection and/or pneumonia treated with C/A, we found no evidence that adding a companion agent improved 30-day survival after adjustment for baseline differences. This study contributes to the literature in three specific ways: it focuses exclusively on critically ill patients, it examines two severe infection phenotypes of major clinical relevance in this setting (bloodstream infection and pneumonia), and it complements mortality analysis with an adapted DOOR framework to provide a broader, patient-centred assessment of treatment benefit. In the IPTW-adjusted analysis, the survival probability ratio for combination versus monotherapy was 1.21 (95% CI: 0.80–1.45), while the DOOR analysis showed only a modest, non-significant probability of a more favourable overall outcome with combination therapy (54.7%). Together, these findings suggest that routine combination therapy is unlikely to provide a clinically meaningful benefit in this setting. At any rate, the study was not prospectively powered, and the sample size limits the precision of causal estimates. Based on the observed event rate in the monotherapy arm (19.5%), the enrolled sample (*n* = 77 vs. *n* = 46) would be adequate to detect only large absolute differences in 30-day mortality (approximately 22–26% at 80% power, two-sided α = 0.05) — an effect size that is arguably implausible for a companion agent added to an already active regimen. Accordingly, the wide confidence intervals surrounding all effect estimates should be interpreted as reflecting substantial residual uncertainty rather than confirmation of equivalence, and our findings should be considered hypothesis-generating pending adequately powered prospective data.

When outcomes were summarized using the weighted DOOR categories, the two strategies produced very similar distributions. Combination therapy was associated with a slightly lower weighted proportion of death (20.4% vs. 29.8%) but a higher proportion of survival with relapse (9.7% vs. 2.6%), resulting in comparable rates of survival without relapse (69.9% vs. 67.6%). This pattern is consistent with an overall effect close to the null and substantial statistical uncertainty, and it highlights that survival alone may not fully capture the trade-offs of different treatment approaches in critically ill populations.

The higher relapse signal observed in the combination group should be interpreted cautiously. In unadjusted analyses, relapse occurred more often with combination therapy (17.4% vs. 3.9%), and the weighted DOOR distribution also suggested a higher frequency of survival with relapse. Several non-mutually exclusive explanations are plausible: confounding by indication (the combination is preferentially used in patients perceived as higher risk), differences in infection phenotype and source control complexity, and longer treatment exposure in the combination group.

Our findings are broadly consistent with the most representative available evidence suggesting no clear survival advantage of routine C/A-based combination therapy over monotherapy once confounding is considered. In a large multicentre cohort, Tumbarello et al. [25] reported nearly identical 30-day mortality with C/A monotherapy and combination therapy, while Oliva et al. [8] found no overall mortality benefit for the addition of fosfomycin in patients with KPC-Kp bloodstream infections, despite some signal for reduced subsequent infectious complications. Likewise, the recent meta-analysis by Hsu et al. [9] found no significant difference in 30-day mortality between combination therapy and monotherapy, although combination regimens were associated with a borderline increase in microbiological eradication. These findings support the interpretation that “combination therapy” is not a homogeneous exposure and that timing, infection site, and partner drug choice likely contribute to the heterogeneity across studies [26].

At the same time, some reports suggest that selected high-risk phenotypes may derive benefit from combination strategies. In particular, Liu et al. [10] observed improved short-term clinical and microbiological outcomes in CRKP pneumonia, although the 30-day mortality difference did not reach statistical significance. Similarly, Zheng et al. [27] reported lower mortality with combination therapy in a small cohort of critically ill patients, although residual confounding cannot be excluded. By contrast, broader evidence remains more neutral: Lai et al. [28] found an apparent mortality disadvantage for monotherapy in CRE infections overall, but this signal was attenuated in analyses restricted to C/A-based regimens. Taken together, current evidence and IDSA guidance [1] support a tailored rather than routine use of combination therapy, reserving companion agents for selected scenarios rather than applying them systematically.

Strengths of this study include the multicentre ICU setting, the explicit causal estimand using a counterfactual framework, and adjustment for measured confounding through inverse probability weighting. The use of DOOR complements mortality endpoints by incorporating relapse into an ordinal patient-centred outcome.

Limitations include the retrospective design and the possibility of residual confounding, including factors not fully captured in the available dataset, such as nuances of source control and microbiological characteristics, despite IPTW creating a balanced pseudo-population in which only the APACHE II score remained slightly above the standardized mean difference threshold of 0.1. Additional limitations derive from the retrospective multicentre design: detailed susceptibility data for adjunctive companion agents were not systematically retrievable for all combination-treated patients, exact tigecycline dosing was not uniformly available, and species-level characterization of co-pathogens in polymicrobial infections was not collected in a standardized fashion across centres. Moreover, the exact agent-specific susceptibility methods for colistin and fosfomycin could not be uniformly reconstructed from the available records. Heterogeneity in companion regimens, together with incomplete data on the timing and duration of concomitant overlap, also raises the possibility of time-dependent exposure bias. Subgroup sample sizes were limited, overall precision was modest, and relapse characterization was constrained by incomplete time-to-relapse data and repeat cultures. We also acknowledge a practical positivity violation within the pneumonia-only subgroup (*n* = 9), in which no patient received monotherapy in the observed data. The marginal survival estimates for this stratum presented in Fig. 2 therefore rely entirely on model-based extrapolation and do not reflect a direct observed comparison, substantially limiting their interpretability and precluding causal claims for this subgroup. In addition, combination therapy comprised companion agents with substantially different mechanisms of action, pharmacokinetic profiles, and presumed levels of in vitro activity against KPC-Kp. Classifying these biologically and clinically distinct regimens under a single “combination therapy” label introduces exposure heterogeneity that may attenuate or obscure treatment effects specific to individual agents. In this respect, we performed a sensitivity analysis restricted to the most frequent combination regimen (C/A plus fosfomycin). Finally, post-treatment emergence of C/A resistance was not systematically assessed, as follow-up microbiological surveillance was not standardized across centres, precluding a reliable comparison of resistance emergence between monotherapy and combination therapy. Early clinical improvement was defined as previously reported to facilitate cross-study comparison; however, we acknowledge that biomarker-based criteria (procalcitonin and CRP reduction) may not always align with patient-centred clinical outcomes, and that the “any one criterion” threshold introduces heterogeneity in what constitutes improvement.

From a clinical perspective, these data support C/A monotherapy as a reasonable default approach for critically ill patients with KPC-Kp infections when adequate source control and optimized dosing are ensured. Combination therapy may still be considered in selected high-risk scenarios, but our findings do not support routine addition of a companion agent to improve 30-day outcomes.

In this context, TDM-guided optimization of C/A monotherapy to achieve specific joint PK/PD targets could potentially eliminate the need for combination therapy. This precision approach ensures microbiological success while avoiding the toxicity and lack of clinical benefit often associated with routine companion agents [29].

Prospective studies with standardized companion regimens and careful accounting for treatment timing are needed to define which patients, if any, derive incremental benefit from combination strategies.

## Supplementary Information

Below is the link to the electronic supplementary material.

Supplementary Material 1

Supplementary Material 2

## Data Availability

The datasets generated during and/or analysed during the current study are not publicly available due to hospitals policies but are available from the corresponding author on reasonable request.
