# Supplementary material for: Ceftazidime-avibactam as monotherapy or in combination for targeted treatment of KPC-producing Klebsiella pneumoniae infections in ICUs: a comparative analysis through counterfactual framework and desirability of outcome ranking
Source: Eur J Clin Microbiol Infect Dis. 2026 May 5;45(8):2541–52. doi: 10.1007/s10096-026-05529-x (PMC13428722; doi:10.1007/s10096-026-05529-x)
Supplement: Supplementary file 1 — Supplementary Material 1 [file 10096_2026_5529_MOESM1_ESM.docx]

**Details of methods (supplementary)**

***Counterfactual Framework and Causal Identification***

To estimate the causal effect of combination antibiotic therapy (ceftazidime-avibactam [C/A] plus additional agent) compared to monotherapy (C/A alone) on 30-day mortality, we employed a counterfactual framework. This approach assumes three key identifying conditions: consistency (i.e., the treatment received corresponds to the treatment of interest), stable unit treatment value assumption (SUTVA, meaning one patient's treatment does not affect another's outcome), and no unmeasured confounding conditional on observed baseline covariates [1].

*Conditional exchangeability (no unmeasured confounding)*

We justify the plausibility of this assumption through the comprehensive set of baseline covariates included in the propensity score model, which was designed to capture the key clinical determinants of the decision to prescribe combination therapy. These include: illness severity at infection onset (APACHE II score, presence of septic shock), requirements for organ support (mechanical ventilation, renal replacement therapy, ECMO), infection phenotype (BSI vs. pneumonia, polymicrobial nature), comorbidity burden (onco-hematologic disease, cardiovascular disease, chronic kidney disease, cirrhosis), and microbiological context (KPC rectal colonization status). This covariate set reflects the principal axes of confounding by indication documented in the literature and observable in our own data, where combination therapy was preferentially prescribed to more severely ill patients with more complex infection profiles. We acknowledge, however, that conditional exchangeability is inherently unverifiable in observational data. In particular, unmeasured factors — such as attending physician preference, institutional protocols, or real-time clinical deterioration not captured by baseline scores — may have influenced treatment allocation and cannot be fully accounted for. This is explicitly acknowledged as a limitation of the study.

*No interference (SUTVA)*

The stable unit treatment value assumption requires both that each patient's potential outcomes are unaffected by other patients' treatment assignments, and that there is a single, well-defined version of each treatment level. The former condition is plausibly met in this setting: patients were treated in distinct ICU episodes and there is no credible epidemiological pathway by which one patient's receipt of combination therapy could alter another's outcome. We therefore consider the no-interference component of SUTVA to be satisfied.

*Positivity*

Positivity requires that each patient has a non-zero probability of receiving either treatment, conditional on their covariate values. While the overall propensity score distribution showed meaningful overlap across the two treatment arms — supporting positivity at the population level — we acknowledge a practical positivity violation within the pneumonia-only subgroup (n=9), in which no patients received monotherapy in the observed data. As a consequence, the survival estimates for this stratum in Figure 2 are derived entirely from model-based extrapolation within the weighted framework, rather than from directly observed comparisons, and should be interpreted with considerable caution. We have added an explicit warning to the Figure 2 legend and to the Limitations section. The pneumonia-only subgroup estimate is therefore presented as exploratory and hypothesis-generating only, and no causal claims are made for this stratum. This finding also reinforces the well-recognised clinical reality that pneumonia cases in critically ill patients are almost universally treated with combination regimens, making monotherapy comparisons in this subpopulation inherently difficult to conduct in observational data.

*Consistency*

The consistency assumption requires that the treatment received corresponds to a well-defined intervention — that is, that "combination therapy" denotes a sufficiently homogeneous exposure. We acknowledge that this assumption is potentially challenged in our study, as combination therapy encompassed companion agents with meaningfully different mechanisms of action, pharmacokinetic profiles, and levels of in vitro activity against KPC-Kp. We address this in two ways. First, we explicitly acknowledge this heterogeneity as a study limitation. Second, we present a sensitivity analysis restricted to C/A + fosfomycin — the dominant and most biologically homogeneous companion regimen in this cohort — as the closest approximation of a consistent exposure definition.

We estimated average treatment effects (ATEs) to provide population-level contrasts between treatment groups. These marginal effects represent the difference in outcomes under the hypothetical scenario in which all patients received combination therapy versus all patients received monotherapy, while holding other factors constant. ATEs were chosen because they are more generalizable to target populations than conditional effects and are less sensitive to model misspecification in the treatment assignment mechanism [2].

Recognizing that Cox proportional hazards models yield conditional hazard ratios rather than marginal causal estimands, we implemented g-computation to derive marginal survival curves. This approach allows us to estimate clinically interpretable survival probability ratios that reflect population-average treatment effects.

Propensity Score Estimation and Inverse Probability of Treatment Weighting

To address confounding by indication and minimize group imbalances, we applied inverse probability of treatment weighting (IPTW) to create a balanced pseudo-population [3]. This method reweights observations such that the weighted population mimics what would be observed in a randomized trial, where treatment assignment is independent of baseline characteristics.

We estimated propensity scores—defined as the conditional probability of receiving combination therapy given observed covariates—using a logistic regression model. The model included the following baseline covariates selected based on their association with both treatment assignment and outcomes: age, sex, body mass index ≥30 kg/m², comorbidities (chronic obstructive pulmonary disease, chronic kidney disease, diabetes mellitus, cardiovascular disease, onco-hematologic disease, cirrhosis), APACHE II score at infection onset, infection acquisition setting (community-acquired versus healthcare-associated), mechanical ventilation use, extracorporeal membrane oxygenation use, renal replacement therapy, infection type, polymicrobial nature of infection, and rectal colonization status with drug-resistant organisms.

Covariate balance was rigorously assessed using standardized mean differences (SMDs) between treatment groups before and after weighting. SMDs exceeding 0.10 in absolute value were considered indicative of substantial imbalance requiring attention [3]. To ensure adequate treatment group overlap and positivity, propensity scores were trimmed at the 1st and 99th percentiles, excluding patients with extreme propensity scores. Weights were stabilized using the marginal probability of treatment to improve efficiency and reduce variance in effect estimates.

***Outcome Model Specification and Non-linearity Assessment***

Within the weighted pseudo-population, we fitted a Cox proportional hazards model with time to death or censoring at 30 days as the outcome. The model incorporated stabilized IPTW weights and was adjusted for all baseline covariates used in the propensity score estimation. This doubly-robust approach provides additional control for residual confounding and improves precision when covariate-outcome associations are strong, even if the propensity score model is slightly misspecified.

To account for potential non-linear relationships between continuous covariates and the hazard of death, we transformed age and APACHE II score using natural splines with 2 degrees of freedom. Internal knots were placed at the quartiles of each variable's distribution [4]. Partial effects plots were generated to visualize the estimated marginal contribution of these spline terms to the hazard while adjusting for all other covariates. These plots help identify thresholds or inflection points in the covariate-outcome relationship.

Multicollinearity among covariates was assessed using the adjusted Generalized Variance Inflation Factor (aGVIF). As a diagnostic rule, aGVIF values exceeding 2 were considered cause for concern and would prompt consideration of variable reduction or combination.

***Marginal Survival Curves and Survival Probability Ratios***

To translate conditional hazard ratios from the Cox model into marginal causal estimands, we employed g-computation [5]. We generated model-based survival predictions for each patient under both treatment scenarios (combination therapy and monotherapy) across the 30-day follow-up period. These individual-level predictions were then averaged across the entire weighted population to obtain marginal survival probabilities with 95% confidence intervals estimated via non-parametric bootstrapping (1000 resamples).

Survival probability ratios (SPRs) were calculated at clinically relevant time points—7, 14, and 30 days—by dividing the marginal survival probability in the combination therapy group by that in the monotherapy group [6]. SPRs greater than 1 indicate survival benefit with combination therapy, while values less than 1 suggest harm. These ratios provide easily interpretable effect measures that directly quantify relative survival improvement.

Stratified sensitivity analyses were conducted by infection type (bloodstream infection, pneumonia, urinary tract infection, and others) to assess the robustness and consistency of treatment effects across clinical subgroups.

***Desirability of Outcome Ranking (DOOR) Analysis***

To provide a comprehensive assessment of clinical success beyond mortality alone, we performed a Desirability of Outcome Ranking (DOOR) analysis following current methodological guidance [7]. We constructed a three-level ordinal scale ranking outcomes from most to least desirable: (1) alive at 30 days without relapse of infection; (2) alive at 30 days with documented clinical relapse; and (3) dead by 30 days. This hierarchy captures both survival and infection-related morbidity.

The DOOR distribution was estimated within the IPTW-weighted pseudo-population to ensure comparisons were balanced for baseline disease severity and comorbidities. We calculated weighted frequencies and percentages of patients in each DOOR category for both treatment groups. To quantify the probability of achieving a better DOOR ranking with combination therapy, we fitted a weighted proportional odds logistic regression model accounting for IPTW weights. The resulting odds ratio represents the odds of being in a more desirable DOOR category for patients receiving combination therapy versus monotherapy.

We also performed sequential dichotomization analysis by collapsing the three-level DOOR ranking into binary categories at each possible threshold. This involved comparing proportions of patients achieving "desirable" (Rank ≤ *k*) versus "undesirable" (Rank > *k*) outcomes for each threshold *k*, providing additional granularity in understanding treatment effects across outcome severity gradients.

***Partial Credit Analysis***

To reflect diverse clinical priorities and patient perspectives, we conducted partial credit analysis based on three distinct scenarios as exemplified elsewhere [8]:

- Scenario A (Survival-Centric): This scenario prioritizes hospital survival above all, assigning full credit only to survival and zero credit to death, essentially treating DOOR as a binary mortality endpoint. It reflects situations where survival is the predominant concern regardless of complications.
- Scenario B (Event-Free Prioritization): This highly risk-averse perspective assigns full credit only to survival without any adverse events (relapse), treating any complication as equivalent to failure. It represents patients or clinicians who prioritize complication-free recovery as equally important to survival itself.
- Scenario C (Balanced Clinical Utility): This scenario employs a weighted approach that prioritizes survival as the primary goal while meaningfully accounting for morbidity associated with non-fatal adverse events. It reflects a pragmatic clinical perspective that values both quantity and quality of survival.

For each scenario, mean partial credit scores were calculated by treatment group and compared. Differences between groups were evaluated with 95% confidence intervals; scenarios where the confidence interval crossed zero indicated no statistically significant difference between treatments under that particular value weighting.

**References**

1. Dahabreh, I.J.; Bibbins-Domingo, K. Causal Inference About the Effects of Interventions From Observational Studies in Medical Journals. *JAMA* **2024**, *331*, 1845–1853, doi:10.1001/jama.2024.7741.

2. Arel-Bundock, V.; Greifer, N.; Heiss, A. How to Interpret Statistical Models Using Marginaleffects for R and Python. *Journal of Statistical Software* **2024**, *111*, 1–32, doi:10.18637/jss.v111.i09.

3. Benedetto, U.; Head, S.J.; Angelini, G.D.; Blackstone, E.H. Statistical Primer: Propensity Score Matching and Its Alternatives†. *Eur J Cardiothorac Surg* **2018**, *53*, 1112–1117, doi:10.1093/ejcts/ezy167.

4. Lopez-Ayala, P.; Riley, R.D.; Collins, G.S.; Zimmermann, T. Dealing with Continuous Variables and Modelling Non-Linear Associations in Healthcare Data: Practical Guide. *BMJ* **2025**, *390*, e082440, doi:10.1136/bmj-2024-082440.

5. Denz, R.; Klaaßen-Mielke, R.; Timmesfeld, N. A Comparison of Different Methods to Adjust Survival Curves for Confounders. *Stat Med* **2023**, *42*, 1461–1479, doi:10.1002/sim.9681.

6. Uno, H.; Claggett, B.; Tian, L.; Inoue, E.; Gallo, P.; Miyata, T.; Schrag, D.; Takeuchi, M.; Uyama, Y.; Zhao, L.; et al. Moving beyond the Hazard Ratio in Quantifying the Between-Group Difference in Survival Analysis. *J Clin Oncol* **2014**, *32*, 2380–2385, doi:10.1200/JCO.2014.55.2208.

7. Hamasaki, T.; He, Y.; Wu, Q.; Howard-Anderson, J.; Boucher, H.W.; Doernberg, S.B.; Holland, T.L.; Powers, J.H.; Wang, J.; Diao, G.; et al. A Patient-Centric Paradigm and Tool for Clinical Research: The DOOR Is Open. *Antimicrob Agents Chemother* **2026**, *70*, e0147825, doi:10.1128/aac.01478-25.

8. Lodise, T.P.; Min, J.; Nathanson, B.H.; Yücel, E. Comparison of Early Treatment with Ceftolozane/Tazobactam versus Polymyxin-Based Therapy of Pneumonia Due to MDR Pseudomonas Aeruginosa (PUMA). *Antimicrob Agents Chemother* **2025**, *69*, e0056925, doi:10.1128/aac.00569-25.
