# Supplementary material for: Ceftazidime-avibactam as monotherapy or in combination for targeted treatment of KPC-producing Klebsiella pneumoniae infections in ICUs: a comparative analysis through counterfactual framework and desirability of outcome ranking
Source: Eur J Clin Microbiol Infect Dis. 2026 May 5;45(8):2541–52. doi: 10.1007/s10096-026-05529-x (PMC13428722; doi:10.1007/s10096-026-05529-x)
Supplement: Supplementary file 2 — Supplementary Material 2 [file 10096_2026_5529_MOESM2_ESM.docx]

**Supplementary Figure 1**. Covariate balance before and after implementation of inverse probability of treatment weighting.


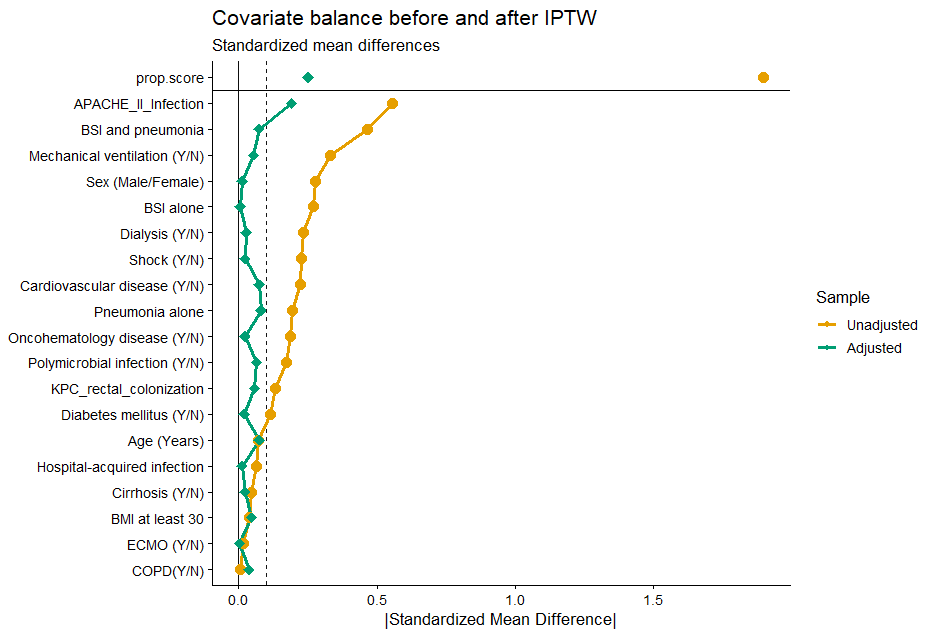


**Supplementary Figure 2**. Partial effects plots regarding continuous variable in the Cox model.

| 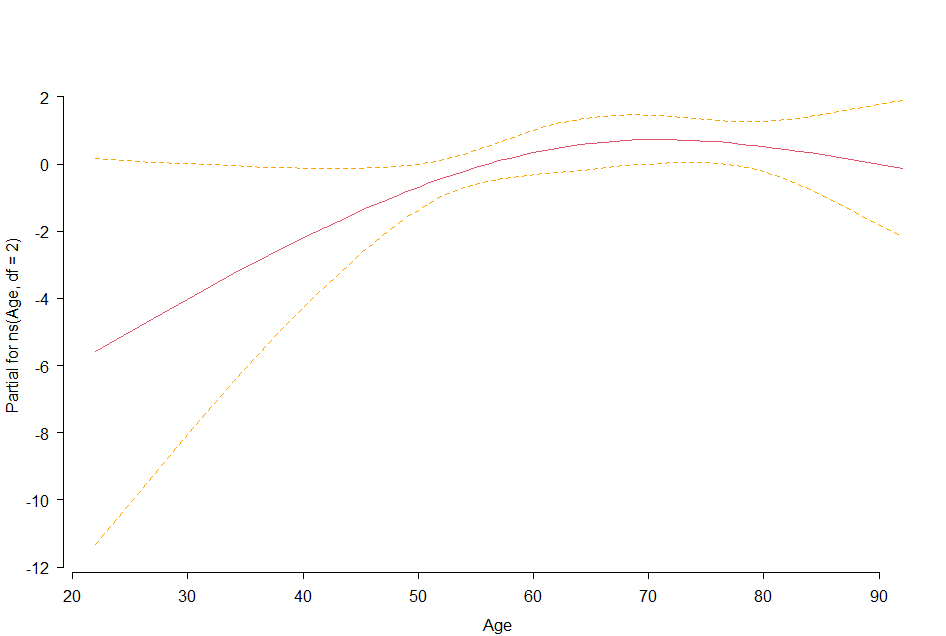 |
| --- |
| 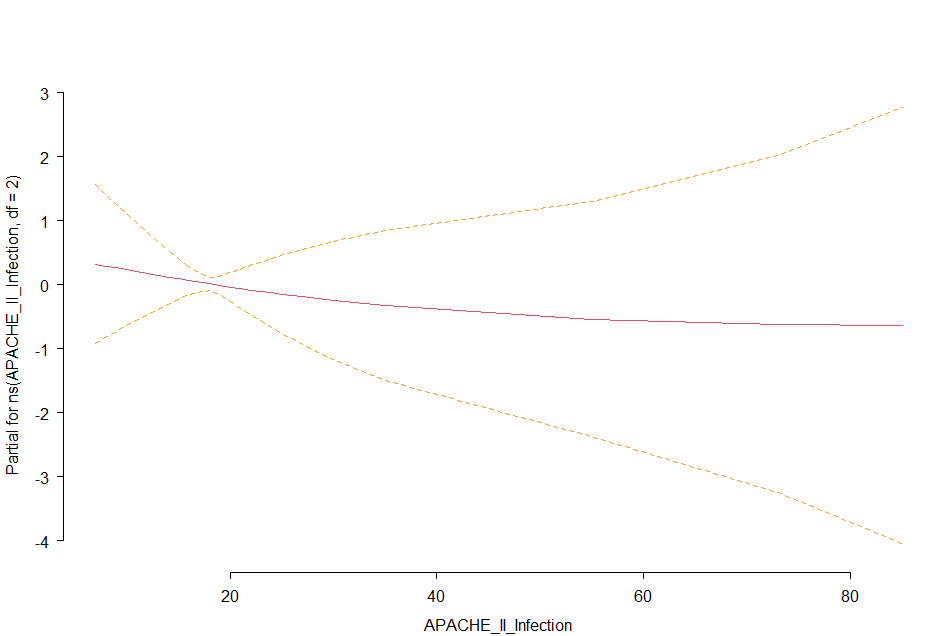 |

**Supplementary Figure 3**. Forest plot of outcomes stratified by combination therapy or not in the desirability of outcome ranking (DOOR) analysis.

**
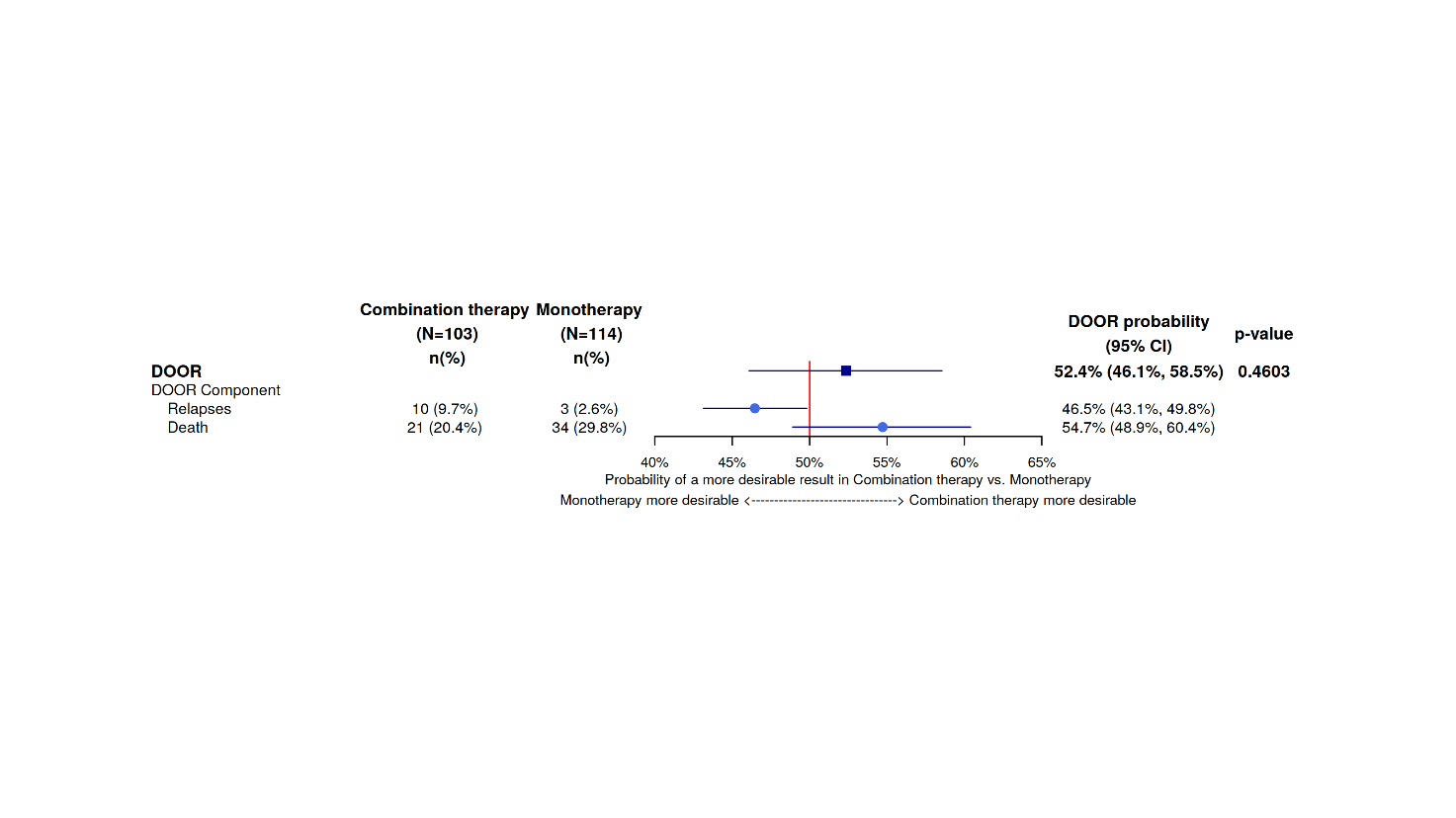
**

**Supplementary Table 1**. Generalized Variance Inflation Factor (GVIF) results.

| **Variable** | **GVIF** | **Df** | **GVIF1/(2⋅Df)** |
| --- | --- | --- | --- |
| Combination therapy | 2.423821 | 1 | 1.556863 |
| Sex | 1.814981 | 1 | 1.347212 |
| ns(Age, df = 2) | 2.215917 | 2 | 1.220080 |
| Body mass index at least 30 | 2.314086 | 1 | 1.521212 |
| Chronic Obstructive Pulmonary Disease | 1.694491 | 1 | 1.301726 |
| Oncohematological disease | 2.174767 | 1 | 1.474709 |
| Diabetes mellitus | 1.772995 | 1 | 1.331539 |
| Cardiovascular disease | 1.419469 | 1 | 1.191415 |
| Cirrhosis | 1.868287 | 1 | 1.366853 |
| ns(APACHE II score, df = 2) | 5.004528 | 2 | 1.495687 |
| Hospital acquired infection | 3.783667 | 1 | 1.945165 |
| Mechanical ventilation | 1.681376 | 1 | 1.296679 |
| ExtraCorporeal Membrane Oxygenation | 1.396351 | 1 | 1.181673 |
| Dialysis | 2.783004 | 1 | 1.668234 |
| Septic shock | 1.650067 | 1 | 1.284549 |
| KPC rectal colonization | 2.512398 | 1 | 1.585055 |
| Type of infection | 4.201337 | 2 | 1.431683 |
| Polymicrobial infection | 2.428216 | 1 | 1.558273 |

Note: in case of you have degrees of freedom (Df) greater than 1, the reference is the last column (GVIF^{1/(2 * Df)}). Thresholds: A common rule of thumb is that a value in the last column greater than 2 (which is equivalent to a standard VIF of 4) might indicate collinearity issues.

**Supplemental Table 2.** Survival probability ratios (SPR) when restricting the analysis to the combination between ceftazidime/avibactam (C/A) and fosfomycin versus monotherapy

| **Time (days)** | **Survival probability % — C/A + Fosfo** | **Survival probability % — Monotherapy** | **SPR** | **95% CI** |
| --- | --- | --- | --- | --- |
| 7 | 87.2% | 84.1% | 1.04 | 0.79–1.22 |
| 14 | 82.4% | 76.9% | 1.07 | 0.76–1.28 |
| 30 | 70.8% | 61.4% | 1.15 | 0.74–1.42 |

**Supplemental Table 3.** Survival probability ratios (SPR) after including time to active treatment in the model.

| **Time (days)** | **Survival probability % — Combination therapy** | **Survival probability % — Monotherapy** | **SPR** | **95% CI** |
| --- | --- | --- | --- | --- |
| 7 | 87.1% | 92.6% | 0.940 | 0.80–1.07 |
| 14 | 82.3% | 87.8% | 0.937 | 0.77–1.08 |
| 30 | 66.5% | 55.3% | 1.202 | 0.73–1.67 |

**Supplemental Table 4.** Desirability of outcome ranking (DOOR) partial credit scoring in scenarios A, B, and C.

| Door Rank | Scenario A (full credit) | Scenario B (no credit) | Scenario C (partial credit) |
| --- | --- | --- | --- |
| Alive and no events | 100 | 100 | 100 |
| Alive with relapse | 100 | 0 | 70 |
| Dead | 0 | 0 | 0 |
